# Esterification Mechanism of Bagasse Modified with Glutaric Anhydride in 1-Allyl-3-methylimidazolium Chloride

**DOI:** 10.3390/ma10080966

**Published:** 2017-08-18

**Authors:** Huihui Wang, Wei Chen, Xueqin Zhang, Chuanfu Liu, Runcang Sun

**Affiliations:** 1 State Key Laboratory of Pulp and Paper Engineering, South China University of Technology, Guangzhou 510640, China; wang.huihui@mail.scut.edu.cn (H.W.); geogeo_chen@163.com (W.C.); xueqin0228@gmail.com (X.Z.); rcsun3@bjfu.edu.cn (R.S.); 2 Beijing Key Laboratory of Lignocellulosic Chemistry, Beijing Forestry University, Beijing 100083, China

**Keywords:** bagasse, ionic liquid, glutaric anhydride, esterification mechanism

## Abstract

The esterification of bagasse with glutaric anhydride could increase surface adhesion compatibility and the surface of derived polymers has the potential of immobilizing peptides or proteins for biomedical application. Due to its complicated components, the esterification mechanism of bagasse esterified with glutaric anhydride in ionic liquids has not been studied. In this paper, the homogenous esterification of bagasse with glutaric anhydride was comparatively investigated with the isolated cellulose, hemicelluloses, and lignin in 1-allyl-3-methylimidazolium chloride (AmimCl) to reveal the reaction mechanism. Fourier transform infrared (FT-IR) indicated that the three components (cellulose, hemicelluloses, and lignin) were all involved in the esterification. The percentage of substitution (PS) of bagasse was gradually improved with the increased dosage of glutaric anhydride (10–40 mmol/g), which was primarily attributed to the increased esterification of cellulose and hemicelluloses. However, the PS fluctuation of lignin led to a decrease in the PS of bagasse at high glutaric anhydride dosage (50 mmol/g). The esterification reactivity of bagasse components followed the order of lignin > hemicelluloses > cellulose. The esterification mechanism was proposed as a nucleophilic substitution reaction. Nuclear magnetic resonance (NMR) analysis indicated that lignin aliphatic hydroxyls were prior to be esterified, and primary hydroxyls were more reactive than secondary hydroxyls in cellulose and hemicelluloses.

## 1. Introduction

The challenges caused by the depletion of fossil fuel sources and the volatility of oil prices has lead to the increasing pressure on the next generation of industry [1]. To cope with the pressure, it is imperative to manufacture renewable materials, transportation fuels and chemicals to supplement or replace those derived from petroleum [2]. As the sustainable alternative with low cost and great renewability, biomass resources draw significant attention to the potential manufacturing biofuels and chemicals, especially lignocellulose biomass. Distributing in many kinds of plants, lignocellulose is mainly composed of carbohydrate polymers (cellulose and hemicelluloses) and polyphenol-based lignin [3]. It is insoluble in conventional solvents due to the complicated structures and the complex linkages among different components, thus limiting its application and making it underutilized. Challenges associated with the insolubility can be resolved by the derivatization of lignocellulose. Esterification is one of the most common derivatization reactions of lignocellulose. Until now, heterogeneous esterification of lignocellulose has been investigated for several decades [4]. The acylation reagent could only react with the hydroxyls on lignocellulose surface, consequently resulting in nonuniform product. Besides, heterogeneous processes are associated with limitations including side reactions, longer time, corrosion and appreciable degradation [5,6].

As the alternative procedure to heterogeneous processes, homogeneous esterification of lignocellulose in appreciate media is still subject of ongoing research. It not only provides the opportunities to obtain the effective control of the percentage of substitution (PS), and distribution and uniformity of the functional groups, but also creates more options to induce novel and functional groups [7]. Currently, the dissolution of lignocellulose has been accomplished in dimethyl sulfoxide (DMSO)/N-methylimidazole, DMSO/lithium chloride, ionic liquids (ILs), DMSO/tetrabutylammonium fluoride, and so on [8,9,10]. Compared with other solvents, ILs have received special attention due to the negligible vapor pressure, thermal and chemical stability, and designability [11]. Among various ILs, imidazium-based ILs can effectively dissolve lignocellulose and allow efficient homogeneous synthesis of lignocellulose esters [12,13]. Homogeneous acetylation of Norway spruce was investigated in three imidazium-based ILs, including 1-butyl-3-methylimidazolium chloride (BmimCl), 1-allyl-3-methylimidazolium chloride (AmimCl), and 1-benzyl-3-methylimidazolium chloride (BnmimCl) [14].

In 2010, the esterification of wood with octanoyl chloride in BmimCl was also reported, and the weight percent gain of products ranged from 121.5% to 298.5% at 130 °C for 6 h [15]. The esterification of lignocellulose with linear anhydride or acyl chloride produces the corresponding carboxylic acid or HCl as byproducts [16]. By contrast, the esterification of lignocellulose with cyclic carboxylic anhydride is an effective derivatization process, and various functional groups can be attached onto lignocellulose. Chemical modification with glutaric anhydride could especially increase hydrophility, hygroscopicity, and surface adhesion compatibility, and the surface of derived polymers has the potential of immobilizing peptides or proteins for biomedical application [17,18]. However, the detailed reaction and degradation behaviors of lignocellulose esterified with glutaric anhydride in the homogenous system are complex due to the complicated mixture of cell wall components. Although some simple investigations have been reported [19,20], there are few studies on the homogeneous esterification mechanism of lignocellulose esterified with glutaric anhydride.

In the present study, cellulose, hemicelluloses and lignin were isolated from bagasse and esterified with glutaric anhydride in AmimCl under the same conditions as bagasse to elucidate the mechanism. Nuclear magnetic resonance (NMR) spectroscopy (i.e., ^1^H NMR, ^13^C NMR, ^31^P NMR, two-dimensional (2D) heteronuclear single quantum coherence (HSQC)), gel permeation chromatograph (GPC), Fourier transform infrared (FT-IR), X-ray photoelectron spectroscopy (XPS) and high performance anion exchange chromatography were used to investigate the physicochemical properties of samples.

## 2. Results and Discussion

### 2.1. Esterification of Bagasse

**Figure 1:**
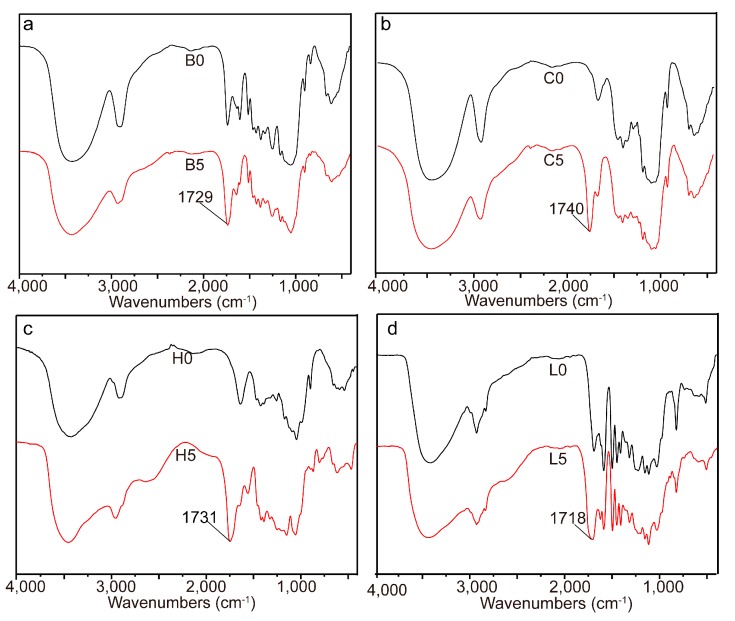
FT-IR spectra of bagasse (**a**); cellulose (**b**); hemicelluloses (**c**); and lignin (**d**) samples.

The homogeneous esterification of bagasse with glutaric anhydride was confirmed by FT-IR studies. The intensity of the band at 1729 cm^−1^ (Figure 1a), corresponding to C=O stretching, increased due to the esterification of bagasse [21]. Due to the complex components and complicated linkages in bagasse, it was very difficult to identify the exact changes of bagasse during the homogeneous esterification. Therefore, the three main components (cellulose, hemicelluloses, and lignin) were isolated from bagasse and esterified with glutaric anhydride under the same conditions as bagasse in AmimCl to elucidate the possible structural changes of bagasse.

Compared with that of unmodified cellulose, the FT-IR spectrum of the esterified cellulose presented a new band at 1740 cm^−1^ (Figure 1b), indicating the formation of cellulose ester in the homogeneous system [16]. Similarly, in the FT-IR spectrum of the esterified hemicelluloses (Figure 1c), the new absorption at 1731 cm^−1^ suggested the esterification of hemicelluloses with glutaric anhydride. By comparison with unmodified lignin, the intensity of the absorbance at 1718 cm^−1^ in the esterified lignin (Figure 1d) increased obviously, indicating the attachment of glutaryl groups onto lignin. These results indicated that cellulose, hemicelluloses, and lignin were all involved in the esterification with glutaric anhydride during the homogenous esterification of bagasse in AmimCl.

### 2.2. PS of Bagasse

The decrease in the hydroxyl content of esterified sample was due to the substitution of glutaryl groups. As shown in Table 1, raising the dosage of glutaric anhydride from 10 to 40 mmol/g resulted in an increase in the substituted hydroxyl content of bagasse samples from 1.64 (B1) to 4.48 (B4) mmol/g, corresponding to a PS increase from 11.47% to 31.33%. A further increase in the dosage of glutaric anhydride from 40 to 50 mmol/g resulted in an unexpected decrease in the substituted hydroxyl content from 4.48 (B4) to 3.96 (B5) mmol/g, corresponding to a PS decrease from 31.33% to 27.69%. These results implied that changing glutaric anhydride dosage could regulate PS of bagasse samples, consistent with the previous study [22]. As shown in Table 2, unmodified bagasse was insoluble in water and common organic solvents, while glutaric anhydride could be dissolved in the selected solvents. After modification, the esterified bagasse samples were insoluble, swollen or soluble in water or organic solvents, especially showing the good solubility in DMSO. These changes of solubility were probably caused by the substitution of hydroxyls in bagasse (with different DS) with glutaryl group, indicating the occurrence of the esterification between bagasse and glutaric anhydride. However, it is impossible to obtain the PS changes of each component during the homogeneous esterification of bagasse because of the complicated components and complex linkages. Therefore, the isolated fractions were esterified with glutaric anhydride at different dosages (10–50 mmol/g) as bagasse to comparatively study the correlation between PS of each component and the dosage of glutaric anhydride.

With the increment of glutaric anhydride dosage from 10 to 50 mmol/g, the substituted hydroxyl content of cellulose samples increased from 1.30 (C1) to 4.52 (C5) mmol/g, corresponding to an increase of PS from 7.02% to 24.41%. These results were probably due to the fact that the increased dosage of glutaric anhydride would enhance the interaction between glutaric anhydride and cellulose [16].

An increase in the dosage of glutaric anhydride from 10 to 50 mmol/g also led to the increased substituted hydroxyl contents of hemicelluloses samples from 1.13 (H1) to 4.54 (H5) mmol/g, corresponding to the PS increase from 7.46% to 29.97%. Considering the similarity between the anhydroglucose units (AGU) and anhydroxylose units (AXU), these increases were also resulted from the enhanced interaction between hemicelluloses and glutaric anhydride.

As shown in Table 1, improving the dosage of glutaric anhydride from 10 to 20, 30, 40, and 50 mmol/g, the substituted hydroxyl contents of lignin samples reached 2.41 (L1), 2.27 (L2), 2.48 (L3), 3.15 (L4), and 2.62 (L5) mmol/g, respectively, and the corresponding PS changed from 47.53% to 44.77%, 48.92%, 62.13%, and 51.68%, respectively. It was interesting to observe the PS fluctuation of lignin samples with the increment of glutaric anhydride dosage. These results indicated that the PS changes of lignin were distinct from those of carbohydrates (cellulose and hemicelluloses). The detailed content changes of lignin hydroxyl groups during the homogeneous esterification are listed in Table 3. Clearly, the aliphatic and phenolic hydroxyl content decreased from 3.96 (L0) to 1.91 (L1), 2.08 (L2), 1.94 (L3), 1.48 (L4), and 2.05 (L5) mmol/g, and from 1.11 (L0) to 0.75 (L1), 0.72 (L2), 0.65 (L3), 0.44 (L4), and 0.40 (L5) mmol/g, respectively. These results indicated both aliphatic and phenolic hydroxyls were esterified with glutaric anhydride during the homogeneous esterification [23]. The ratio of aliphatic hydroxyls to phenolic hydroxyls obviously decreased from 3.57 (L0) to 2.55 (L1), indicating the prior esterification of aliphatic hydroxyls at very low dosage of glutaric anhydride(10 mmol/g). With the increment of glutaric anhydride dosage from 10 to 50 mmol/g, the ratio gradually increased from 2.55 (L1) to 5.13 (L5), which was due to the increased esterification of phenolic hydroxyls.

### 2.3. Reactive Sites of Bagasse

**Figure 2:**
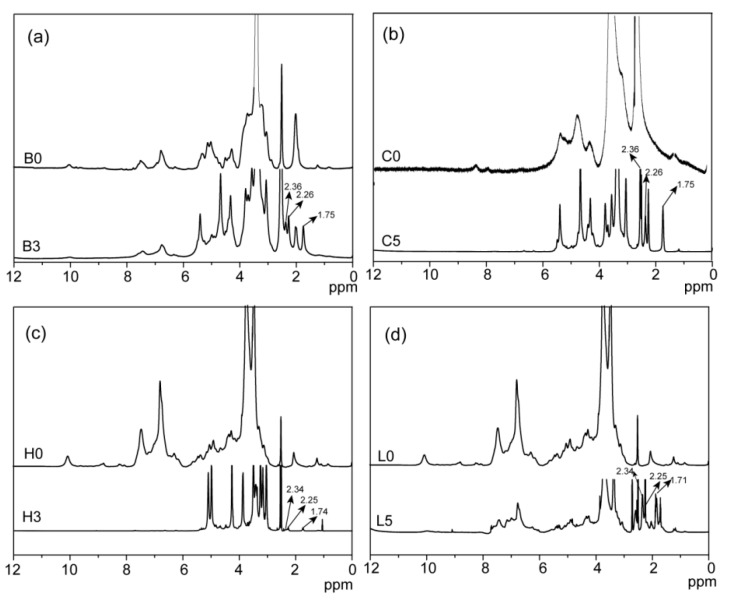
^1^H NMR spectra of bagasse (**a**); cellulose (**b**); hemicelluloses (**c**) and lignin (**d**) samples.

**Figure 3:**
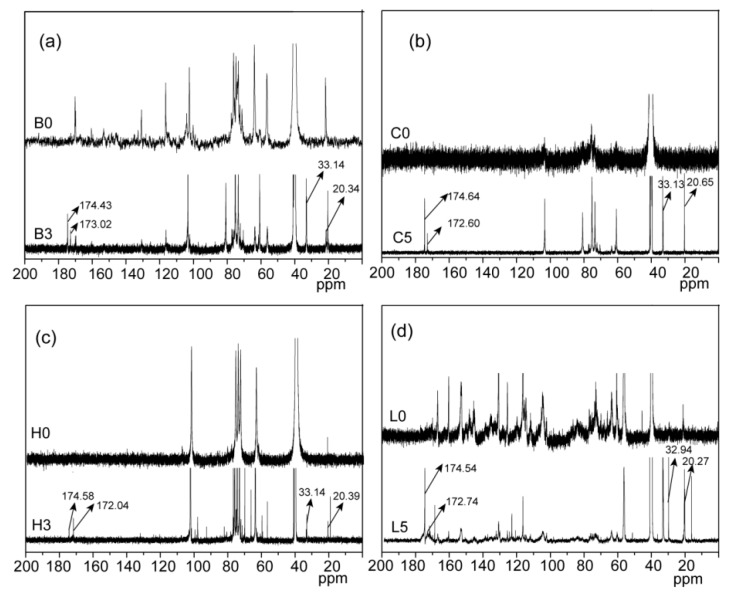
^13^C NMR spectra of bagasse (**a**); cellulose (**b**); hemicelluloses (**c**) and lignin (**d**) samples.

1D (^1^H, ^13^C) and 2D NMR (HSQC) spectroscopy are powerful technologies to confirm the occurrence of esterification, and analyze the detailed reactive sites of bagasse during the homogeneous esterification. In the ^1^H NMR spectrum of the esterified bagasse (Figure 2a), peaks at 1.75, 2.26, and 2.36 ppm for glutaryl protons proved the esterification of bagasse with glutaric anhydride. Compared with that of unmodified bagasse, peaks at 20.34, 33.14, 173.02, and 174.43 ppm in ^13^C NMR spectrum of the esterified bagasse B3 (Figure 3a) correspond to glutaryl carbons, further confirming the esterification between bagasse and glutaric anhydride, consistent with the FT-IR analysis.

**Figure 4:**
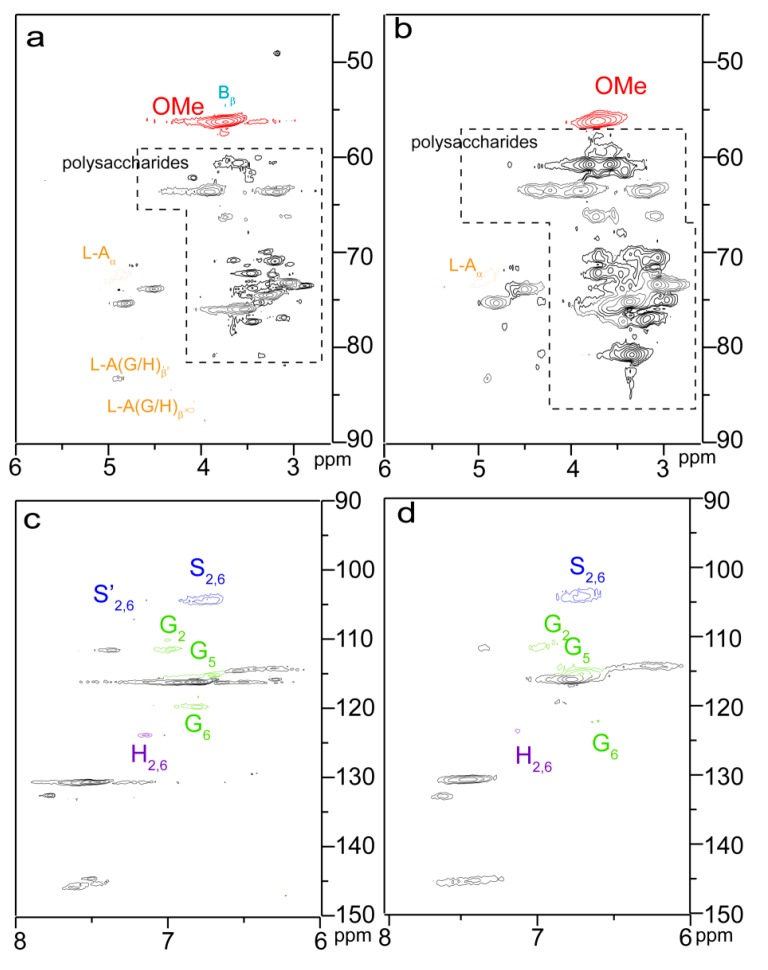
2D HSQC spectra of unmodified (B0, **a**,**c**) and esterified (B3, **b**,**d**) bagasse.

In the 2D HSQC NMR spectra of bagasse samples (Figure 4), cross-peaks from carbohydrate polymers (cellulose and hemicelluloses) were seriously overlapped, and the partial cross-peaks from lignin were recognizable. The poor distinguishability of the correlation signals in the HSQC spectra of bagasse samples made it inaccurate to analyze the detailed esterification information of reactive sites in each component during the homogenous esterification. Therefore, the detailed esterification information of reactive sites in each component was further analyzed with the HSQC spectra of the esterified fractions.

**Figure 5:**
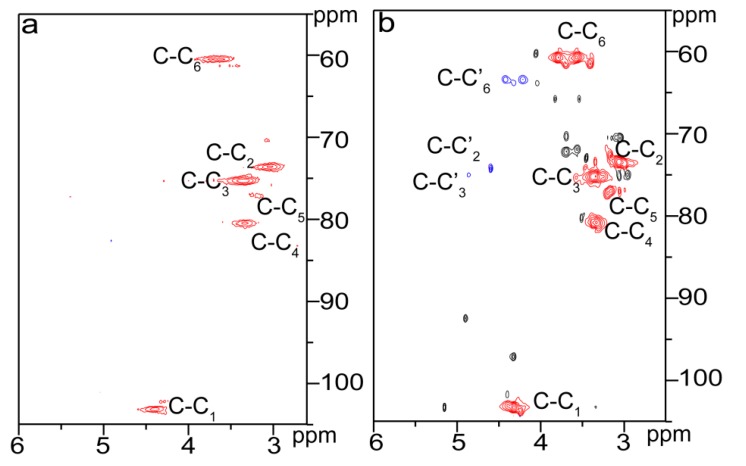
2D HSQC NMR spectra of unmodified (C0, **a**) and esterified (C5, **b**) cellulose.

Compared with that of unmodified cellulose, proton peaks from the glutaryl group at 1.75, 2.26, and 3.36 ppm in the ^1^H NMR spectrum of the esterified cellulose (Figure 2b) were clearly identified. Besides, carbon signals from glutaryl group in the ^13^C NMR spectrum of the esterified cellulose (Figure 3b) at 20.65, 33.13, 172.60, and 174.64 ppm were also well resolved. These results confirmed the attachment of glutaryl group onto cellulose during the homogeneous esterification. The HSQC spectra of unmodified (C0, a) and esterified (C5, b) cellulose in DMSO-*d*_6_ are shown in Figure 5. The primary polysaccharide correlation peaks were well assigned based on their 1D (^1^H and ^13^C) NMR spectra and the previous literature [24,25]. In the HSQC spectrum of cellulose sample C5, the correlation peaks at δ_C_/δ_H_ 63.40/4.42 and 63.43/4.21 ppm correspond to C’_6_/H’_6_ from substituted C_6_ (C–C’_6_) in AGU, and the cross-peaks at δ_C_/δ_H_ 74.24/4.60 and 74.99/4.86 ppm are for C’_2_/H’_2_ and C’_3_/H’_3_ from substituted C_2_ (C–C’_2_) and C_3_ (C–C’_3_) in AGU, respectively. The successful esterification of hydroxyls at C_2_, C_3_, and C_6_ positions of AGU during the homogeneous derivatization was also observed in the esterification of cellulose with succinic anhydride in DMSO/BmimCl [26]. The PS of C_2_–OH, C_3_–OH, and C_6_–OH of AGU could be easily evaluated upon the integral area of the substituted and unsubstituted characteristic correlations [25]. The results showed that the PS of hydroxyls at C_2_, C_3_, and C_6_ positions of AGU were 2.2%, 0.7% and 10.7%, respectively. Therefore, the reactivity of hydroxyls in AGU during the homogeneous esterification followed the order of C_6_–OH > C_2_–OH > C_3_–OH.

Considering the short-branched chain structure consisting of various sugar units in hemicelluloses [27], the reactivity of main-chains and side-chains of hemicelluloses during the homogeneous esterification was further evaluated. According to the contents of different monosaccharides, main-chains of hemicelluloses were xylan, and side-chains were consisted of the other monosaccharides. As shown in Table 4, the monosaccharide content of side-chains obviously decreased from 12.17% (H0) to 6.17% (H1), suggesting the prior esterification of side-chains at a very low dosage of glutaric anhydride (10 mmol/g), which was probably due to the more reactive primary hydroxyl groups on side chains [25]. Further increasing glutaric anhydride dosage from 10 to 40 mmol/g led to an increase in the monosaccharide content of side-chains from 6.17% (H1) to 8.63% (H4), indicating the primary esterification of secondary hydroxyls on main-chains, which was probably due to the abundance of main-chain sugars. An increase in the dosage of glutaric anhydride from 40 to 50 mmol/g resulted in a decrease in the monosaccharide content of side-chains from 8.63% (H4) to 5.73% (H5) and an increase in the content of xylose from 91.37% (H4) to 94.27% (H5). These results indicated that secondary hydroxyls on side-chains were more easily esterified than those on main-chains, which was probably due to the lower steric hindrance of side-chains.

**Figure 6:**
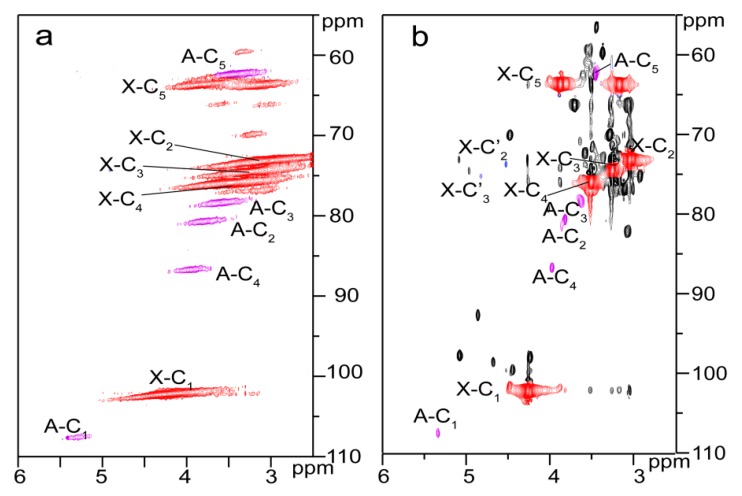
2D HSQC NMR spectra of unmodified (H0, **a**) and esterified (H3, **b**) hemicelluloses.

Three peaks at 1.74, 2.25, and 2.34 ppm in the ^1^H NMR spectrum of the esterified hemicelluloses (Figure 2c) are assigned to the glutaryl protons, and signals at 20.29, 33.13, 172.04, and 174.58 ppm in the ^13^C NMR spectrum of the esterified hemicelluloses (Figure 3c) are originated from the carbons of glutaryl group. These confirmed the occurrence of esterification between hemicelluloses and glutaric anhydride in AmimCl. The reactive sites of AXU from xylan during the homogeneous esterification were explored with 2D HSQC NMR in DMSO-*d*_6_, as shown in Figure 6. The characteristic cross-peaks for xylan and arabinose were well resolved in the spectra of hemicelluloses samples (H0 and H3), based on their 1D (^1^H and ^13^C) NMR spectra and the previous study [28]. The correlation peaks at δ_C_/δ_H_ 74.21/3.87 and 76.01/4.25 ppm are associated with C’_2_/H’_2_ and C’_3_/H’_3_ from the substituted hydroxyls at C_2_ (X-C’_2_) and C_3_ (X-C’_3_) positions of AXU. By integrating the correlations assigned to substituted and unsubstituted C_2_ and C_3_ in AXU, the PS of hydroxyls at C_2_ and C_3_ positions of AXU were 1.7% and 1.6%, indicating the similar reactivity of secondary hydroxyls of AXU.

**Figure 7:**
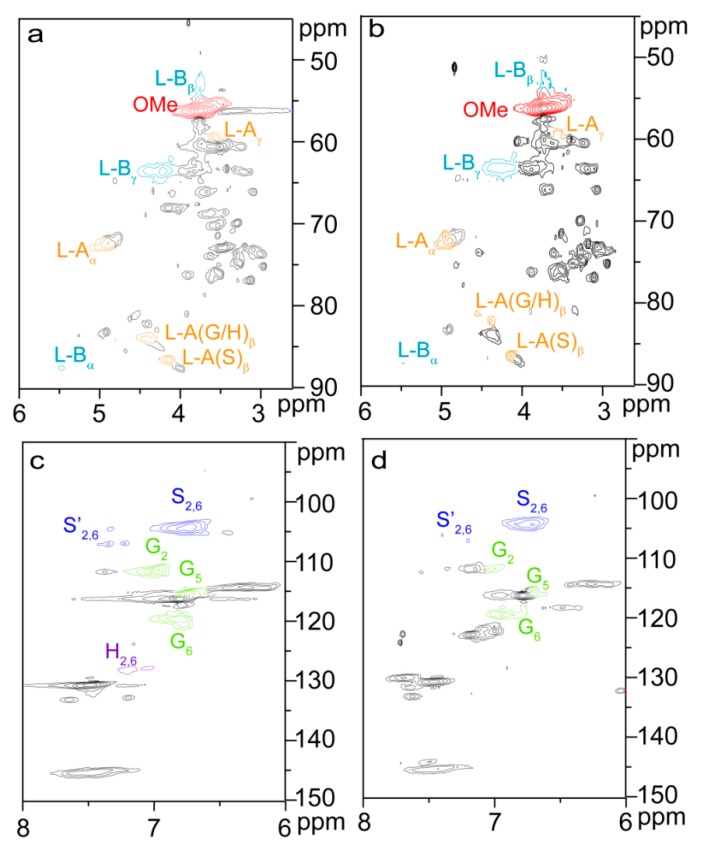
2D HSQC NMR spectra of unmodified (L0, **a**,**c**) and esterified (L5, **b**,**d**) lignin.

**Figure 8:**
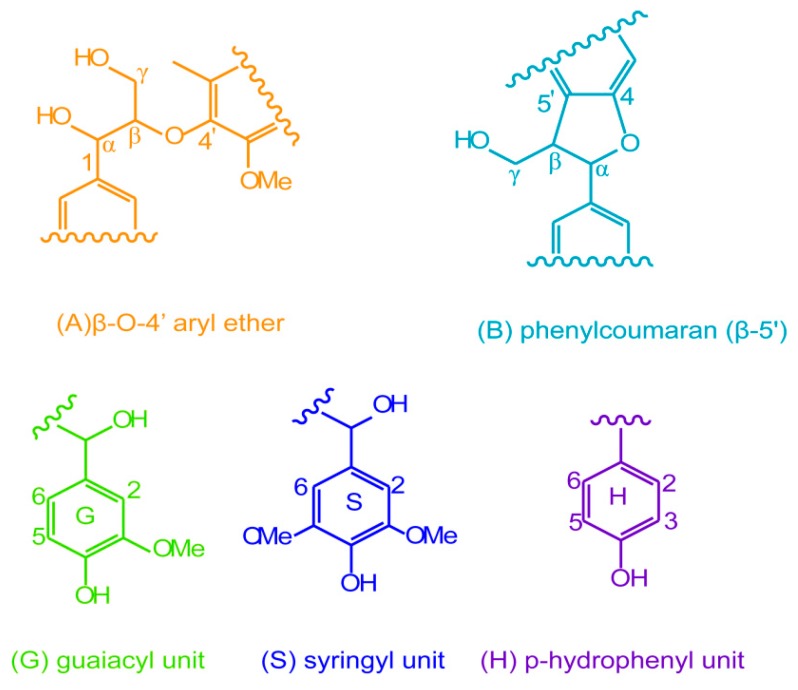
The substructures and aromatic units identified in lignin.

By comparison, three signals at 1.71, 2.25, and 2.34 ppm in the ^1^H NMR spectrum of the esterified lignin (Figure 2d) correspond to the glutaryl protons, and four carbon signals at 20.27, 32.94, 172.74, and 174.54 ppm from the ^13^C NMR spectrum of the esterified lignin (Figure 3d) relate to the carbons of glutaryl group. The presence of these signals confirmed the esterification between lignin and glutaric anhydride under the selected conditions. Semiquantitative 2D HSQC analysis of lignin samples were used to reveal the content changes of lignin substructures, according to the previous literature [24,29]. The HSQC spectra of unmodified (L0, **a** and **c**) and esterified (L5, **b** and **d**) lignin are shown in Figure 7, and the substructures and aromatic units identified in lignin are depicted in Figure 8. By semiqualitative analysis, the content of primary lignin substructures is listed in Table 5. The content of β–*O*–4′ aryl ether decreased from 45.6/100Ar (L0) to 44.4/100Ar (L5), suggesting the cleavage of β–*O*–4′ during the homogeneous esterification [30]. After esterification, cross-peaks from phenylcoumaran (β-5′) and H-units could not be distinguished under the present contour level, indicating the degradation of β-5′ and H-units during the homogeneous esterification. Besides, the syringyl to guaiacyl (S/G) ratio slightly increased from 1.11 (L0) to 1.16 (L5), which was probably resulted from the slight degradation of G-units during the homogeneous esterification. Semiquantitative 2D HSQC analysis revealed that the cleavage of lignin side-chains and degradation of aromatic units simultaneously occurred during the homogeneous esterification. The cleavage of lignin side-chain linkages and the degradation of aromatic units were probably due to the presence of glutaric acid, which was released from glutaric anhydride in AmimCl [31].

### 2.4. XPS Analysis of Bagasse Samples

**Figure 9:**
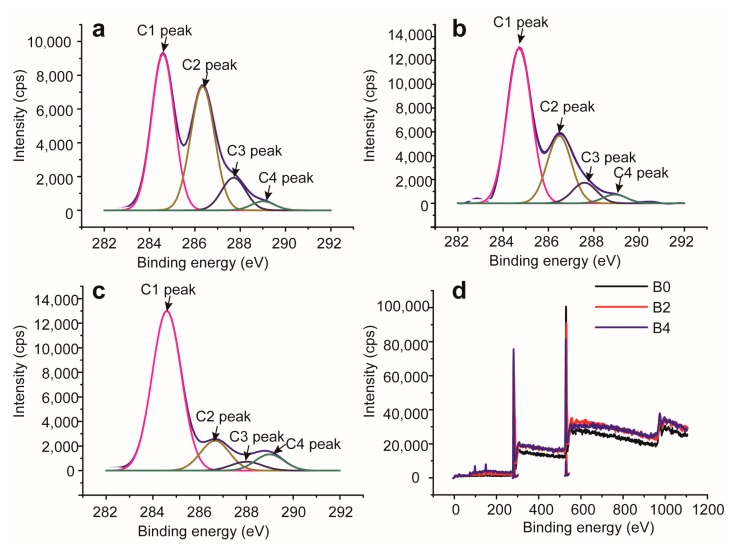
C1s spectra of unmodified (B0, **a**) and modified (B2, **b**; B4, **c**) bagasse samples, and the general XPS spectra (**d**) of bagasse samples.

Figure 9 illustrates the deconvoluted C1s signals of unmodified (B0) and modified (B2 and B4) bagasse samples. As reported in the previous literature [32], the C1s peak was deconvoluted into four subpeaks: C1 peak corresponds to C–C or C–H; C2 and C3 peaks refer to the C–O and C=O or O–C–O, respectively; C4 peak is assigned to O–C=O. Compared with that in B0, the intensity of C4 peak increased obviously as C2 peak decreased strongly in B2 and B4, which was due to the increased O–C=O bonds from the new formed esters. This suggested the successful esterification between bagasse and glutaric anhydride. In addition, the XPS spectra and elemental analysis of bagasse samples are shown in Figure 9d and Table 6, respectively. As shown in Figure 9d, there were no new elements in the esterified bagasse samples, indicating no introduction of impurities into the esterified bagasse samples. The introduction of glutaric groups could increase the carbon to oxygen (C/O) ratio. As shown in Table 6, the C/O ratio increased from 1.88 (B0) to 2.53 (B2), and further to 2.94 (B4), with the gradual increase of PS of bagasse samples. These results confirmed the esterification of bagasse with glutaric anhydride, as well as the increment of the PS of bagasse samples with the increased dosage of glutaric anhydride. These results were consistent with the PS results, FT-IR and NMR analysis.

### 2.5. Degradation of Esterified Bagasse

The degradation degree of bagasse during the homogeneous esterification was further investigated with GPC analysis. The weight-average molecular weight (*M*_w_) decreased from 42,129 (B0) to 35,511 (B3) g/mol (Table S1), suggesting the degradation of bagasse after the homogeneous esterification. Besides, compared with that of unmodified bagasse, some signals disappeared in the HSQC spectrum of the esterified bagasse B3 (Figure 4), confirming the degradation of bagasse during the homogeneous esterification. However, because of the complicated components and complex linkages in bagasse, it was impossible to directly obtain the detailed degradation information of each component. Therefore, the detailed degradation information of each component was illuminated with the GPC analysis of the esterified fractions.

The noticeably increased correlation peaks from low-molecular fractions in the HSQC spectrum of cellulose sample C5 indicated the significant degradation of cellulose upon esterification in AmimCl. As shown in Table S1, the *M*_w_ of cellulose samples obviously decreased from 57,787 (C0) to 35,511 (C5) g/mol, confirming the degradation of cellulose during the homogeneous esterification. Compared with unmodified hemicelluloses H0, signals from low-molecular fractions also obviously increased in the HSQC spectrum of esterified hemicelluloses, suggesting the degradation of hemicelluloses during the homogeneous esterification. GPC results showed that an improvement in the dosage of glutaric anhydride from 0 to 40 mmol/g also led to the *M*_w_ decrease from 34,116 (H0) to 24,493 (H4) g/mol, affirming the degradation of hemicelluloses samples. The degradation of carbohydrates (cellulose and hemicelluloses) was probably caused by glutaric acid formed upon esterification in AmimCl [25], which was similar to the previous report [33]. After esterification, the *M*_w_ of lignin samples increased from 15,105 (L0) to 29,554 (L5) g/mol, confirming the successful introduction of glutaryl groups into lignin [34]. The *M*_w_ of lignin sample L5 was much higher than the theoretical value calculated from the PS of esterified lignin, which was possibly due to the diesterification of lignin at high glutaric anhydride dosage.

### 2.6. Possible Esterification Mechanism of Bagasse Modified with Glutairc Anhydride

**Figure 10:**
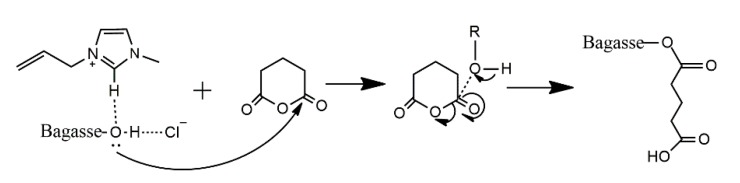
The proposed esterification mechanism of bagasse modified with glutaric anhydride in AmimCl.

Based on the above-mentioned results and the previous studies [35,36], a possible esterification mechanism of bagasse modified with glutaric anhydride in AmimCl was proposed as a nucleophilic substitution in essence, as illustrated in Figure 10. In the homogeneous system, because of the presence of electron-sufficient oxygen, hydroxyl groups of bagasse can easily attack glutaric anhydride, leading to the intermediate. Due to the strong electrophilic ability of carboxyl and carbonyl groups of glutaric anhydride, the ring of the intermediate can be easily opened, resulting in the glutarylated bagasse. Because of the presence of carboxyl groups on the attached glutaryl groups, other free hydroxyls of bagasse allow the further esterification with the attached glutaryl group, forming the diesterified bagasse.

## 3. Materials and Methods

### 3.1. Materials

Bagasse was provided by a local factory (Jiangmen, China). It was air-dried and ground to prepare 20–40 mesh size particles (450–900 µm). The dried, ground bagasse was extracted with toluene-ethanol (2:1, *v/v*) for 4 h and air-dried in a cabinet oven at 50 °C for 24 h. According to our previous study [25], the contents of cellulose, hemicelluloses, and lignin in bagasse were 44.85%, 33.13%, and 19.24%, respectively.

AmimCl was purchased from Shanghai Cheng Jie Chemical Co. Ltd. (Shanghai, China). Cellulase (4–10 units/mg) was purchased from Sigma-Aldrich (Shanghai, China). Glutaric anhydride and other chemicals with analytical grade were purchased from Guangzhou Chemical Reagent Factory (Guangzhou, China).

### 3.2. Isolation of Cellulose, Hemicelluloses, and Lignin from Bagasse

Cellulose, hemicelluloses and lignin were isolated from the extractive-free bagasse, as per our previous publication [37].

### 3.3. Homogeneous Esterification with Glutaric Anhydride in AmimCl

The ball-milling of extractive-free bagasse or cellulose (12.0 g) was performed on a planetary BM4 ball-miller (Grinder, Beijing, China) at 220 rpm for 4 h. The isolated hemicelluloses and lignin were directly used without ball-milling. The prepared materials (0.5 g) were dispersed in 10 g of AmimCl on a magnetic stirrer without heating under N_2_ atmosphere. The suspension was heated at 90 °C for 4 h under agitation to obtain a clear solution. Glutaric anhydride was added to the solution in proportion (10–50 mmol/g). The reaction was performed at 90 °C for 90 min with stirring under the protection of N_2_. The product was precipitated with 200 mL of 99 wt % ethanol and filtered out. The solid residues were washed with ethanol (three times, total 600 mL) to eliminate AmimCl, unreacted glutaric anhydride and byproducts, and freeze-dried.

### 3.4. Determination of PS

According to our previous report [37], the back-titration method were used to determine the substituted hydroxyl contents of bagasse, cellulose, and hemicelluloses samples, and the theoretical hydroxyl contents of unmodified bagasse, cellulose, and hemicelluloses were 14.30, 18.52, and 15.15 mmol/g.

The hydroxyl contents of lignin samples were calculated from ^31^P NMR spectroscopy, as the reported literature [31].

The PS of sample was calculated by Equation (1).

(1) $$PS=\frac{n_{\mathrm{OH}\prime }}{n_{\mathrm{OH}}}\times 100\%$$

where *n*_OH_ (mmol/g) is the hydroxyl content of unmodified sample, *n*_OH’_ (mmol/g) is the substituted hydroxyl content of esterified sample, and *PS* is the percentage of the substitution.

### 3.5. Solubility of Bagasse Samples

Samples (10 mg) was added in water and different organic solvents (0.5 mL), and stirred for 12 h. The selected organic solvents included DMSO, DMF, and acetone.

### 3.6. Characterization

The physic-chemical properties of samples were characterized with FT-IR (Bruker, Karlsruhe, Germany) and NMR (^1^H NMR, ^13^C NMR and ^1^H-^13^C HSQC NMR, Bruker Advance III 600M, Bruker, Karlsruhe, Germany), as the previous literature [37].

The composition of neutral sugars and uronic acid of samples was determined with high performance anion exchange chromatography, according to the reported literature [38].

XPS was conducted on an Axis Ultra DLD (Kratos, Manchester, UK) with Al-Kα radiation (1486.71 eV). The base pressure in the analytical chamber was 5 × 10^−9^ torr. The analysis spot was 700 × 300 μm. The peak positions were corrected for sample charging by setting the C1s binding energy at 284.6 eV.

The *M*_w_ and number-average molecular weights (*M*_n_) of samples were determined by GPC (Waters 1515, Milford, MA, USA) with differential detector (Waters 2414). Samples were previously benzoylated to increase the dissolubility in tetrahydrofuran according to the following procedure [39]. Samples (0.3 g), benzoyl chloride (5 mL) and 2,4-dimethylamino pyridine (0.2 mg) were added to pyridine (5 mL) in a flask. The reaction was performed at 80 °C with stirring for 24 h under N_2_ atmosphere. After the required time, 200 mL of isopropanol was slowly poured into the resulting mixture to stop the reaction. The solid was filtered out, washed thoroughly with isopropanol (three times, total 600 mL) to remove pyridine, unreacted benzoyl chloride and 2,4-dimethylamino pyridine, and then dried at 60 °C in vacuum to a constant weight.

## 4. Conclusions

Cellulose, hemicelluloses and lignin were all involved in the homogeneous esterification of bagasse with glutaric anhydride in AmimCl. The reactivity order of bagasse components followed lignin > hemicelluloses > cellulose. The esterification was proposed as a nucleophilic substitution reaction, which could account for the high reactivity of lignin and reactive sites of bagasse during the homogeneous esterification. The priority on lignin aliphatic hydroxyls was revealed by ^31^P NMR analysis. The reactivity of secondary hydroxyls of hemicelluloses side-chains was higher than those of main-chains, and the reactivity of secondary hydroxyls on AXU was similar. The reactivity order of hydroxyls on AGU followed C_6_–OH > C_2_–OH > C_3_–OH. In addition, the obvious degradation of cellulose and hemicelluloses was responsible for the degradation of bagasse during the homogeneous esterification. This study provided the enhanced understanding of the esterification behaviors of bagasse during the homogeneous esterification with glutaric anhydride, which will facilitate the improved methodology to manufacture the esterified lignocellulosic materials.

## Figures and Tables

**Table 1:** The substituted hydroxyl content and the percentage of substitution of samples.

Samples	Temperature (°C)	Time (Min)	Dosage (mmol/g)	^a^ n’_OH_ (mmol/g)	^b^ PS (%)
B1	90	90	10	1.64	11.47
B2	90	90	20	2.11	14.76
B3	90	90	30	3.21	22.45
B4	90	90	40	4.48	31.33
B5	90	90	50	3.96	27.69
C1	90	90	10	1.30	7.02
C2	90	90	20	1.43	7.72
C3	90	90	30	1.64	8.86
C4	90	90	40	1.61	8.69
C5	90	90	50	4.52	24.41
H1	90	90	10	1.13	7.46
H2	90	90	20	1.49	9.83
H3	90	90	30	1.63	10.76
H4	90	90	40	2.60	17.16
H5	90	90	50	4.54	29.97
L1	90	90	10	2.41	47.53
L2	90	90	20	2.27	44.77
L3	90	90	30	2.48	48.92
L4	90	90	40	3.15	62.13
L5	90	90	50	2.62	51.68

^a^ The content of the substituted hydroxyl groups; ^b^ The percentage of substitution.

**Table 2:** The solubility of bagasse samples in water and common organic solvents.

Samples	B0	Glutaic Anhydride	B1	B2	B3	B4	B5
Water	-	++	+	+	-	-	+
DMSO	-	++	++	++	++	++	++
DMF	-	++	+	+	+	+	++
Acetone	-	++	+	+	+	+	+

++: soluable; +: swollen; -: insoluable.

**Table 3:** The content of hydroxyl groups in lignin samples (L0–L5).

Samples	L0	L1	L2	L3	L4	L5
Aliphatic OH (mmol/g)	3.96	1.91	2.08	1.94	1.48	2.05
Phenolic S–OH (mmol/g)	0.09	0.05	0.04	0.04	0.01	0.09
Phenolic G–OH (mmol/g)	0.26	0.14	0.12	0.10	0.06	0.15
Phenolic H–OH (mmol/g)	0.76	0.56	0.56	0.51	0.37	0.16
Total phenolic hydroxyls (mmol/g)	1.11	0.75	0.72	0.65	0.44	0.40
aliphatic OH/Phenolic OH	3.57	2.55	2.89	2.98	3.36	5.13
COOH (mmol/g)	0.11	0.17	0.18	0.41	0.31	1.03

**Table 4:** The content of neutral sugars (relative % dry weight) and uronic acid (relative % dry weight) in the hemicellulosic samples.

Samples	^a^ Ara (%)	^b^ Gal(%)	^c^ Glc (%)	^d^ Xyl (%)	^e^ Glua (%)	^f^ Total Side-Chain (%)
H0	4.20	0.52	6.01	87.83	1.44	12.17
H1	0	0.84	4.43	93.83	0.89	6.17
H2	0.13	0.79	4.81	93.34	0.94	6.66
H3	1.11	0.76	4.97	92.30	0.86	7.70
H4	1.32	0.96	5.48	91.37	0.86	8.63
H5	4.20	0	1.52	94.27	0	5.73

^a^ Arabinose; ^b^ Galactose; ^c^ Glucose; ^d^ Xylose; ^e^ Glucuronic acid; ^f^ Total content of monosaccharides except xylose.

**Table 5:** Quantitative composition information of lignin samples (L0 and L5) from 2D HSQC NMR spectra.

Samples	L0	L5
Aryl ether (A)	45.6/100Ar	44.4/100Ar
Phenylcoumaran (B)	3.5/100Ar	3.2/100Ar
S/G	1.11	1.16

**Table 6:** The elemental analysis of bagasse samples obtained from XPS spectra.

Samples	B0	B2	B4
O (%)	34.63	28.87	25.35
C (%)	65.37	71.13	74.65
C/O ratio	1.88	2.53	2.94

## Supplementary Materials

The following are available online at www.mdpi.com/1996-1944/10/8/966/s1, Table S1: Weight-average (*M*_w_), number-average (*M*_n_) molecular weight, and polydispersity (*M*_w_/*M*_n_) of the samples.
