# Supplementary material for: Esterification Mechanism of Bagasse Modified with Glutaric Anhydride in 1-Allyl-3-methylimidazolium Chloride
Source: Materials (Basel). 2017 Aug 18;10(8):966. doi: 10.3390/ma10080966 (PMC5578332; doi:10.3390/ma10080966)
Supplement: Supplementary file 1 [file materials-10-00966-s001.pdf]

Esterification Mechanism of Bagasse Modified with Glutaric Anhydride in 1-Allyl-3-methylimidazolium Chloride

Hui-Hui Wang [1], Wei Chen [1], Xue-Qin Zhang [1], Chuan-Fu Liu [1,*], Run-Cang Sun [1,2]

[1] State Key Laboratory of Pulp and Paper Engineering, South China University of Technology, Guangzhou 510640, P. R. China.

[2] Beijing Key Laboratory of Lignocellulosic Chemistry, Beijing Forestry University, Beijing 100083, P. R. China

* Correspondence: chfliu@scut.edu.cn; Tel.: +86-20-87113912

Table S1. Weight-average (M_w), number-average (M_n) molecular weight, and polydispersity (M_w/M_n) of the samples

**Samples**	M_n	M_w	M_w/M_n
B0	39,026	42,129	1.08
B2	36,953	38,965	1.05
B3	35,274	35,511	1.00
C0	56,129	57,787	1.03
C3	41,086	41,359	1.00
C5	34,654	34,788	1.00
H0	33,061	34,116	1.03
H2	32,660	32,800	1.00
H4	23,839	24,493	1.02
L0	10,834	15,105	1.39
L2	14,920	18,616	1.24
L5	24,300	29,554	1.22
